# Alteration of HIV epitope processing and presentation by HIV protease inhibitors

**DOI:** 10.1186/1742-4690-9-S2-P275

**Published:** 2012-09-13

**Authors:** G Kourjian, J Boucau, M Shimada, NY Lai, S Le Gall

**Affiliations:** 1 Ragon Institute of MGH, MIT and Harvard, Boston, MA, USA

## Background

Epitopes displayed by MHC-I come from the multistep degradation of proteins by intracellular peptidases such as proteasome and aminopeptidases or cathepsins in the exogenous pathway. We hypothesize that due to structural homologies HIV protease inhibitors (PIs) used in antiretroviral therapies may affect activities of cellular peptidases involved in epitope processing and may affect epitope presentation to immune cells.

## Methods

Using a fluorogenic assay the effect of 5 HIV-1 PIs (Ritonavir, Saquinavir, Nelfinavir, Indinavir, Atazanavir) on proteasome, aminopeptidase and cathepsin activities was tested in PBMCs from at least 6 healthy donors. Using PBMC cytosol as a source of peptidases and HPLC and mass spectrometry to define and quantify the degradation products, the effect of HIV PIs on HIV peptide processing kinetics and HIV epitope half-life was assessed. Finally we assessed the impact of PIs on the endogenous processing and presentation of epitopes by infected cells to CD8 T cells using a fluorescence-based cytotoxicity assay.

## Results

HIV PIs variably altered proteasome, post-proteasomal aminopeptidases and cathepsin activities. Depending on the PI, some activities were inhibited (from 1.1 to 5 folds, p<0.001), enhanced (1.2 to 9 folds, p<0.001), and others not changed. These PI-induced changes in protease activities modified HIV peptide processing patterns and HIV epitope intracellular half-life prior to MHC-I loading. Depending on the PI and the epitope the half-life was increased 1.5 fold (p<0.01) or decreased 1.3 fold (p<0.05). Furthermore HIV PI altered (from 2.2 fold decrease to 1.3 fold increase, p<0.01) the presentation of HIV epitopes and recognition by epitope-specific CD8 T cells.

## Conclusion

These findings suggest that in HIV-infected patients an antiretroviral therapy including PIs might -by altering host proteases function- modify the pattern of epitope presentation, leading to the elicitation of additional CTL responses against HIV and potentially against other pathogens co-infecting HIV+ persons.

This project was supported by NIH-NIAID grant R01-A1084106.

